# Unexpectedly Low Mutation Rates in Beta-Myosin Heavy Chain and Cardiac Myosin Binding Protein Genes in Italian Patients With Hypertrophic Cardiomyopathy

**DOI:** 10.1002/jcp.22636

**Published:** 2011-02-01

**Authors:** Roberta Roncarati, Michael VG Latronico, Beatrice Musumeci, Stefania Aurino, Annalaura Torella, Marie-Louise Bang, Gloria Saccani Jotti, Annibale A Puca, Massimo Volpe, Vincenzo Nigro, Camillo Autore, Gianluigi Condorelli

**Affiliations:** 1 Instituto di Tecnologie Biomediche (ITB), Consiglio Nazionale delle Ricerche (CNR)Milan, Italy; 2 Scientific and Technology Park, Istituto di Ricovero e Cura a Carattere Scientifico (IRCCS) MultiMedicaMilan, Italy; 3 Department of Cardiology, 2nd Faculty, “La Sapienza” UniversityRome, Italy; 4 Telethon Institute of Genetics and MedicineNaples, Italy; 5 Laboratorio di Genetica Medica, Dipartimento di Patologia Generale, Seconda Università degli Studi di NapoliNaples, Italy; 6 Dulbecco Telethon Institute c/o ITBCNR, Milan, Italy; 7 Department of Public Health, University of ParmaParma, Italy; 8 Dipartimento di MedicinaCNR, Rome, Italy

## Abstract

Hypertrophic cardiomyopathy (HCM) is the most common genetic cardiac disease. Fourteen sarcomeric and sarcomere-related genes have been implicated in HCM etiology, those encoding β-myosin heavy chain (*MYH7*) and cardiac myosin binding protein C (*MYBPC3*) reported as the most frequently mutated: in fact, these account for around 50% of all cases related to sarcomeric gene mutations, which are collectively responsible for approximately 70% of all HCM cases. Here, we used denaturing high-performance liquid chromatography followed by bidirectional sequencing to screen the coding regions of *MYH7* and *MYBPC3* in a cohort (n = 125) of Italian patients presenting with HCM. We found 6 *MHY7* mutations in 9/125 patients and 18 *MYBPC3* mutations in 19/125 patients. Of the three novel *MYH7* mutations found, two were missense, and one was a silent mutation; of the eight novel *MYBPC3* mutations, one was a substitution, three were stop codons, and four were missense mutations. Thus, our cohort of Italian HCM patients did not harbor the high frequency of mutations usually found in *MYH7* and *MYBPC3*. This finding, coupled to the clinical diversity of our cohort, emphasizes the complexity of HCM and the need for more inclusive investigative approaches in order to fully understand the pathogenesis of this disease. J. Cell. Physiol. 226: 2894–2900, 2011. © 2011 Wiley-Liss, Inc.

Hypertrophic cardiomyopathy (HCM) is the most common genetic disease of the myocardium, occurring in about 1:500 individuals in the general population (Maron et al., 1995; Bos et al., 2009). It is characterized by usually asymmetric left ventricular (LV) hypertrophy in the absence any obvious cause, such as hypertension (Chung et al., 2003). The clinical course of HCM is extremely diverse: many patients remain asymptomatic throughout life, others develop symptoms of atrial fibrillation and/or heart failure, and some die suddenly, often at a young age and without previous symptoms—indeed, HCM is the most common cause of sudden cardiac death in athletes (Maron et al., 1980; Alcalai et al., 2008).

HCM is an autosomal dominant disorder and, to date, >500 distinct mutations in 14 genes encoding sarcomeric or sarcomere-related proteins have been implicated in its etiology (Marian and Roberts, 2001; Seidman and Seidman, 2001). However, these account for only 70% of genetically tested HCM patients (Marian, 2001). Recently, two or more sequence alterations present either in the same or in different sarcomeric genes (compound or double heterozygosity) were demonstrated to occur in 3–5% of HCM patients (Ingles et al., 2005; Keren et al., 2008). The presence of multiple gene mutations is often associated with a greater clinical severity of the disease (Felker et al., 2000; Chien, 2003; Ingles et al., 2005). Unfortunately, the relationship between genotype and phenotype is complex for HCM and is still challenging.

The goal of the present study was to identify the genetic variations (mutations and single nucleotide polymorphisms—SNPs) present in the two sarcomeric protein genes that have been reported to account for about 50% of genotyped cases of HCM (Van Driest et al., 2005; Keren et al., 2008), that is, *MHY7*, which encodes β-myosin heavy chain (Geisterfer-Lowrance et al., 1990), and *MYBPC3*, encoding cardiac myosin binding protein (Watkins et al., 1995), and find any correlation between the variation detected and the clinical phenotype presented. To this end, we used denaturing high-performance liquid chromatography (DHPLC) followed by direct sequencing (Sanger) to screen a cohort of Italian patients with HCM.

Unexpectedly, we identified only 6 and 18 distinct mutations, respectively, in *MYH7* and *MYBPC3*—of which, respectively, 3 and 8 were novel—in a total of only 22.4% of our unrelated patients. Moreover, a paucity of significant genotype/phenotype correlations indicated the impossibility of clinically differentiating HCM on the basis of only these two selected sarcomeric genes.

## Materials and Methods

### Clinical evaluation of patients

One hundred thirty-six Italian patients (n = 125 unrelated) diagnosed with HCM between 2005 and 2007 by cardiologists at the dedicated cardiomyopathy outpatient clinic of Azienda Ospedaliera Sant'Andrea, Sapienza University of Rome, were enrolled for the study. The study was approved by the Internal Ethic Committee of Azienda Ospedaliera Sant'Andrea, and all participants gave written informed consent to be included in the study. Patients with any relative diagnosed with HCM were considered familial cases, and patients without a family history of HCM and affected relatives were considered sporadic cases. When carriers were found, screening was suggested for first-degree family members.

All probands and relatives underwent physical examination, 12-lead electrocardiogram, two-dimensional echocardiography, Doppler studies, and 24-h Holter monitoring. The diagnosis of HCM was based on echocardiographic demonstration of a hypertrophied and non-dilated left ventricle (wall thickness ≥15 mm in adults, or the equivalent relative to body surface area in children) in the absence of other cardiac or systemic diseases that could produce a comparable magnitude of LV hypertrophy (Spirito et al., 2000). The greatest thickness measured at any site in the LV wall was considered to represent the maximal wall thickness (Spirito et al., 2000). End-diastolic LV cavity and left atrial dimensions were assessed from the derived M-mode echocardiogram. LV outflow tract obstruction was considered present when the peak instantaneous outflow gradient estimated by continuous wave Doppler was ≥30 mm Hg under basal conditions (Maron et al., 2003; Autore et al., 2005).

### Genetic analysis

#### Sample preparation

Aliquots of 5 ml of peripheral blood were collected in tubes containing EDTA. Genomic DNA was extracted using DNA Qiamp Midi kit (QIAGEN, Crawley, UK) according to the manufacturer's instructions. The suitability for sequencing of genomic DNA was verified spectrophotometrically (Thermo Fisher Scientific, Barrington, IL).

#### PCR primers and exon amplification for DHPLC

Mutational analysis of the exons and exon/intron boundaries (at least 35 intron nucleotides from the junctions) of *MYH7* and *MYBPC3* was carried out with DHPLC. For all samples with an abnormal DHPLC elution profile, the putative sequence anomaly was investigated by automated dye terminator cycle (bidirectional) sequencing. Primer pairs were designed to amplify *MYH7* and *MYBPC3* exon-containing fragments. Primers were compared by the web-based Primer3 program (PRIMER3; primer3_http://www.cgi, v 0.2; http://frodo.wi.mit.edu). Each oligonucleotide was also checked by Blast against the NCBI data bank genome for specificity (BLAST, http://www.ncbi.nlm.nih.govBLAST; NCBI, http://www.ncbi.nlm.nih.gov). Primer sequences are given in Supplementary Tables S1 (*MYH7*) and S2 (*MYBPC3*). PCR reactions were set up in 25 µl containing the following: 15 pmol of each primer, 1× PCR buffer with 1.5 mM magnesium chloride, 200 µM of each deoxynucleotide triphosphate (Amersham, Little Chalfont, UK), 1.25 U of Ampli-Taq Gold DNA polymerase (Applied Biosystems, Foster City, CA), and 60 ng of DNA. The conditions used were as follows: a denaturation step at 95°C for 7 min; 34 cycles at 95°C for 30 sec., annealing at 60°C for 90 sec, and extension at 68°C for 60 sec, adding 3 sec for each cycle; a final extension at 68°C for 10 min. All PCR reactions were carried out in a PCR Express Thermalcycler (Celbio, Milan, Italy). The same annealing temperature for all the exons was used, allowing different exons to be amplified in the same 96/384-well plate. The PCR products were stored at 4°C before DHPLC analysis.

#### Experimental conditions for DHPLC

DHPLC analysis was carried out with the WAVE DNA fragment analysis system, using models 3500A and 3500HT (Transgenomic, Omaha, NE). A PCR size range of 150–450 bp was used in order to reach >95% sensitivity. The PCR mixture (5–8 µl) was injected into a preheated C18 reversed-phase column with non-porous (polystyrene/divinylbenzene) particles (DNASepTM column; Transgenomic). The injected sample was then eluted by a linear acetonitrile gradient consisting of buffer A and buffer B. DNA was detected at 260 nm. Melting curves were predicted using Wavemaker software (Transgenomic), and a comparison of the retention times at different temperatures was performed with control fragments bearing known variations. Each variation was usually detectable in a range of ±1.5°C. The typical analytical gradient time was 3.5 min with the 3500A model and 2.5 min with the 3500HT model. Buffer B concentration was increased at 2% per min.

#### DNA sequencing

The PCR amplicons were purified with Exo-Sap enzymes (Applied Biosystems) and then sequenced. Direct sequencing was performed by automated cycle sequencing with a 3130XL Genetic Analyzer (Applied Biosystems). The sequencing reaction was performed with the following protocol: 2.5 pM/µl for each primer (forward or reverse), 2 µl of Big Dye Terminator mix (Perkin Elmer, Boston, MA) containing dideoxyribonucleotides, deoxyribonucleotides, a Taq polymerase, 5× TACS buffer (400 mM Tris–HCl, pH 8, 10 mM MgCl_2_, and 100 mM SO_4_(NH_4_)_2_, pH 9), 5 µl of DNA (100 ng), and H_2_O to a final volume of 10 µl. At the end of the sequencing reaction the samples were purified on a Sephadex G50 membrane by centrifugation at 1,700 rpm for 2 min; 5 µl of the purified sample thus obtained was added to 10 µl of deionized formamide and denatured for 3 min at 95°C before being subjected to electrophoresis. The electrophoresis apparatus used was an ABI PRISM 3100 automatic sequencer (Applied Biosystems).

### Statistical analysis

All continuous data are given as mean ± SD. Baseline characteristics between two groups were analyzed by Student's *t*-test for continuous variables and by the Fisher exact test for categorical data.

## Results

### Patients

The clinical characteristics, including electrocardiographic and echocardiographic data, of the patients are given in Table 1 (see also Supplementary Table S3). Most of the patients (87.2%) were born in Rome or in other parts of the Lazio region; 16 were from other regions of Italy. Fifty-one had a family history of HCM. The majority of patients (92%) were asymptomatic or mildly symptomatic; 10 had moderate or severe symptoms of heart failure (NYHA class III–IV) and 6 had progressed to the “end-stage phase” of the disease, with impaired LV systolic function (ejection fraction <50%). At echocardiography, 40 patients presented with an LV outflow tract obstruction. In most patients, hypertrophy was localized to the anterior septum, whereas it was apical in only 12. Interventions and events are also given in Table 1. In 19 patients judged to be at high risk of sudden death, a cardioverter-defibrillator (ICD) had been implanted as a primary prevention; 4 of them had already experienced one appropriate intervention (ICD intervention). Two patients experienced non-fatal stroke and another underwent myotomy/myectomy.

**Table 1:** Clinical Features of Unrelated HCM Patients at Time of Genetic Testing

Clinical features	Patients (n = 125)
Men (%)	79 (63.2)
Age at study (yrs)	54.2 ± 16^b^
Age at diagnosis (yrs)	40.7 ± 18^b^
Family history of HCM (%)	51 (40.8)
Family history of SCD (%)	38 (30.4)
NYHA III–IV functional class (%)	10 (8.0)
Chest pain (%)	55 (44.0)
Syncope (%)	19 (15.2)
NSVT (%)	14 (10.9)
Atrial fibrillation^a^ (%)	16 (12.8)
Electrocardiographic analysis	
LVH (%)	79 (63.2)
Q wave (%)	39 (31.2)
T wave inversion (%)	82 (65.6)
Echocardiographic features	
LVOT obstruction (%)	40 (32.0)
Left atrium (mm)	45.5 ± 7^b^
Maximum LVWT (mm)	19.9 ± 5^b^
LVEF (%)	65.1 ± 7^b^
Localization of hypertrophy	
Anterior septum (%)	97 (77.6)
Posterior septum (%)	6 (4.8)
Lateral wall (%)	10 (8.0)
Apical wall (%)	12 (9.6)
Interventions and events	
ICD (%)	19 (15.2)
Myotomy/myectomy (%)	1 (0.8)
Alcohol septal ablation (%)	0 (0)
End stage evolution (%)	6 (4.8)
Stroke (%)	2 (1.6)
ICD intervention (%)	4 (3.2)

Yrs, years; SCD, sudden cardiac death; NYHA III–IV, New York Heart Association class III–IV (classification of heart failure); NSVT, non-sustained ventricular tachycardia; ECG, electrocardiogram; LVH, left ventricular hypertrophy; LVOT, left ventricular outflow tract; LVWT, left ventricular wall thickness; LVEF, left ventricular ejection fraction; ICD, implantable cardioverter defibrillator.

a Either paroxymal or permanent.

b Mean ± SD.

### Genetic analysis

Thirty-two individuals in our cohort of 136 HCM patients (23.5%) were found carrying mutations: of these, 28 were unrelated, accounting for 22.4% of the 125 unrelated patients. Specifically, 6 distinct mutations were found in *MHY7* in 9/125 (7.2%) patients and 18 distinct mutations were found in *MYBPC3* in 19/125 (15.2%) patients (Tables 2 and 3, respectively). All mutations were in heterozygosis. Multiple mutations, present either within the same gene or in both genes contemporaneously, were not encountered. A total of 384 chromosomes from healthy, unrelated Italian subjects were used as controls.

**Table 2:** Variations Detected on MYH7* by DHPLC Followed by Direct Sequencing**

DNA variation	Exon	Type of mutation	Protein mutation	Novel	Patient ID
Panel (a) mutations^a^					
c.1331 A>G	12	Missense	N444S	Yes	154
c.2795 T>A	21	Missense	M932K	No	201
c.4954 G>T	33	Missense	D1652Y	Yes	183
c.4472 C>G	30	Missense	S1491C	No	190^c^, 199^c^, 206, 293
c.1549 C>A	13	Missense	L517M	No	248
c.3153 G>A	23	Silent	A1051A	Yes	273, 227

DNA variation	Exon/intron	Type of SNP	Protein variation	Novel^d^	Patient ID
Panel (b) SNPs					
c.732 C>T	Exon 6	Synonymous^b^	F244F	rs2069542	219, 299, 93, 230, 273, 255
c.597 A>G	Exon 5	Synonymous	A199A	rs2069541	291, 260, 288, 289, 189
c.2967 T>C	Exon 22	Synonymous	I989I	Yes	167, 217
c.4566 T>C	Exon 31	Synonymous^b^	T1522T	Yes	172
c.4193A>T	Exon 29	Non-synonymous	Q1398L	Yes	182
−110 t>c	−110 t>c	5' UTR	—	Yes	162, 95, 165, 242
c3855+27 t>a	Intron 26	—	c.3855+27t>a	Yes	173, 203, 229, 255, 261, 280, 299
c.5655+32 g>a	Intron 36	—	c.5655+32g>a	Yes	173, 203, 280, 284, 290

* Protein: GenBank NP_000248; cDNA: GeneBank NM_000257 Reverse strand.

** Carried out on exons and intron/exon boundaries (from a minimum of 35 up to 50 bases) in 136 subjects.

a All variations were in heterozygosis.

b Potential splicing alteration.

c Daughter and mother.

d SNP ID is given for known SNPs. Novel mutations were absent in the healthy controls (n = 192). The Cardiogenomics and Human Genome Mutation databases were used to assign the status of novel mutation—known mutations were not tested in controls.

**Table 3:** Variations Detected on MYBPC3* With DHPLC Followed by Direct Sequencing**

DNA variation	Exon	Type of mutation	Protein mutation	Novel	Patient ID
Panel (a) mutations^a^					
c.1090G>A	11	Potential splicing alteration	A364T	No	233
c.1813G>C	17	Potential splicing alteration	D605H	No	180^c^, 186^c^, 208^c^
c.1624G>C	15	Potential splicing alteration	E542Q	No	244
c.2309-2A>G	22	Potential splicing alteration	2309−2	No	234
c.2905+1G>A	25	Potential splicing alteration^b^	2905+1	Yes	157
c.2905C>T	25	Nonsense: STOP codon	Q969X	No	188
c.2827C>T	25	Nonsense: STOP codon	R493X	Yes	220
c.3034C>T	27	Nonsense: STOP codon	Q1012X	No	181
c.3697C>T	31	Nonsense: STOP codon	Q1233X	No	251
c.2846–2847InsT	25	Frameshift: STOP codon	M949IfsX100	Yes	278
c.2258–2259InsT	21	Frameshift: STOP codon	K754EfsX78	Yes	195, 228
c.3192–3193InsC	28	Frameshift: STOP codon	K1065QfsX11	No	194
c.2654C>T	24	Missense	T885M	Yes	265
c.2002C>T	19	Missense	R668C	Yes	214
c.3251T>C	28	Missense	L1084P	Yes	177^d^, 185^d^
c. 2311G>A	22	Missense	V771M	No	221
c.2429 G>A	25	Missense	Arg810His	Yes	270
c.1373G>A	14	Missense	R458H	No	268

DNA variation	Exon/intron	SNP	Novel^e^	Protein variation	Patient ID
Panel (b) SNPs					
c.472G>A	Exon 4	Non-synonymous	rs3729986	V158M	270
c.977G>A	Exon 11	Non-synonymous	rs34580776	R326Q	251
c.1564 G>A	Exon 15	Non-synonymous	rs11570082	A522T	221
c.492 C>T	Exon 4	Synonymous	rs3218719	G164G	214
c.2550 C>T	Exon 23	Synonymous	rs3729953	V849V	278, 251, 268
c.3288 G>A	Exon 28	Synonymous	rs1052373	E1096E	234
c.506–12	Intron 4	—	Yes	delC	195, 251, 270
c.1223+29 G>A	Intron 12	Synonymous	rs11570078	—	181, 221, 194
c.2340+18 C>G	Intron 21	Synonymous	rs3729948	—	278, 251 268, 194
c.2737+12 C>T	Intron 24	Synonymous	rs3729936	—	251, 268, 194

* Protein: GenBank NP_000247.2; cDNA: GenBank NM_000256.3 reverse strand.

** Carried out on exons and intron/exon boundaries (from a minimum of 35 up to 50 bases) in 136 subjects.

a All variations were in heterozygosis.

b Possible exclusion of exon 25.

c Related (father, son, and nephew).

d Related (father and son).

e SNP ID is given for known SNPs. Novel mutations were absent in the healthy controls (n = 192). The Cardiogenomics and Human Genome Mutation databases were used to assign the status of novel mutation—known mutations were not tested in controls.

### Mutations in *MYH7*

Six variations present in *MYH7* (4.8%) were distinct mutations that were not found in the control group (Table 2a). Three were novel and carried by 4/125 (3.2%) patients, whereas the other 3/6 were known mutations harbored by 5/125 (4%) patients.

#### Novel mutations

**Fig. 1:**
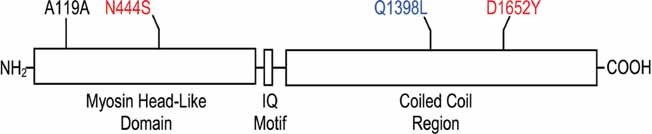
Schematic representation of homosapiens β-myosin heavy chain protein and the location of the novel mutations (n = 3) and non-synonymous SNP found in the 136 HCM patients analyzed. Red, missense mutations; black, silent mutation; blue, non-synonymous SNP.

Two of the mutations found (N444S and D1652Y) were novel missense mutations. A third (A1051A) was a silent mutation harbored by two unrelated patients. Figure 1 gives the location of these novel mutations in a schematic representation of the *MYH7* protein. Electropherograms and chromatograms for the novel mutations found in *MYH7* are given in Supplementary Figure 1.

#### Known mutations

Three of the mutations encountered are already listed in public databases: L517M is a missense mutation that has a pathogenic effect and has been linked to HCM with progression to dilated cardiomyopathy (Nanni et al., 2003); M932K is another missense mutation (Iascone et al., 2007); S1491C (Hougs et al., 2005) was found in four of our subjects (two unrelated patients and a mother/daughter pair).

### Mutations in *MYBPC3*

Eighteen distinct mutations were found in *MYBPC3* (14.4%): five were potential alternative splicing sites (all generated by nucleotide substitutions), seven were stop codons (four caused by nonsense substitutions and three formed from insertions generating frameshifts), and six were missense mutations (Table 3a). Nine (7.2%) unrelated patients harbored novel mutations, whereas 10/125 (8%) carried known mutations. Three of the mutations were present in more than one patient.

#### Novel mutations

**Fig. 2:**
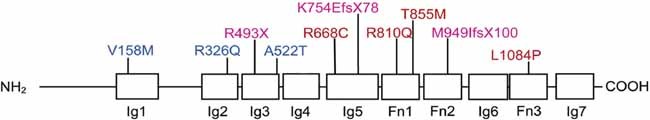
Schematic representation of the location of the novel mutations (n = 8) and the non-synonymous SNPs (n = 3) detected within cardiac myosin binding protein in the 136 HCM patients analyzed. Novel mutation 2905+1 (exon 25) is not shown. Red, missense mutations; purple, nonsense mutations producing stop codons; blue, non-synonymous SNPs.

Eight out of the 18 mutations detected (6.4%) were novel: c.2905+1 was a substitution potentially causing the excision of exon 25 through altered splicing; 3 were mutations that produced premature stop codons, 1 of which (R493X) was caused by a nonsense nucleotide substitution and 2 of which were caused by nucleotide insertions (M949IfsX100 and K754EfsX78); the remaining 4 (T885M, R668C, L1084P, and R810Q) were missense mutations with a probable pathogenic effect caused by the substitution of an amino acid. L1084P was detected in a father/son pair, and the novel insertion K754EfsX782 was carried by two unrelated patients, one of which (patient ID 195) presented also with a delC in intron 4, only 12 bp upstream of exon 5. Figure 2 is a schematic summary of the novel variations recorded. Electropherograms and chromatograms for novel mutations detected in *MYBPC3* are given in Supplementary Figure 2.

#### Known mutations

Ten out of the 18 mutations detected (8%) are already described in public mutation databases: A364T (Smaniotto et al., 2009), D605H (Alders et al., 2003), E542Q (Carrier et al., 1997), and c.2309-2A>G (Niimura et al., 2002) are substitutions potentially generating splicing alterations, the last one found in the intronic side of the junction and D605H harbored by three related patients (father, son, and nephew); three (Q969X, Q1012X, and Q1233X) are nonsense mutations creating stop codons in exons 25, 27, and 31, respectively (Yu et al., 1998; Erdmann et al., 2001; Nanni et al., 2003); K1065QfsX11 (Girolami et al., 2006) is a nucleotide insertion generating, through a frameshift, a premature stop codon in exon 28; V771M (Garcia-Castro et al., 2005); and R458H (Van Driest et al., 2004) are missense mutations caused by amino acid substitutions in exon 22 and 14, respectively (Cardiogenomics Mutation Database, 2005; HGMD, http://www.hgmd.cf.ac.uk/ac/all.php).

### Polymorphisms

In addition to the above mutations, we found several SNPs.

#### MYH7

We found 6 distinct novel SNPs in *MYH7* in 31 patients. All but two of these SNPs were detected in more than one patient (Table 2b): three (I989I, T1522T, and Q1398L) resided in the coding region—except for Q1398L these were synonymous variations; three were nucleotide substitutions present either in the 5'UTR (−110t>c), in intron 26 (c.3855+27t>a), or in intron 36 (c.5655+32g>a). F244F and T1522T were substitutions of the last nucleotide of exons 6 and 31, respectively, causing possible splicing alterations. Only two distinct synonymous SNPs (F244F and A199A) have already been described (http://cardiogenomics.med.harvard.edu/home). At the moment, we can only indicate these genetic alterations as polymorphisms of doubtful etiologic role in that they were not found in 384 chromosomes from control subjects.

#### MYBPC3

Ten different SNPs where found in *MYBPC3* in 21 patients; 5 of these SNPs were found to be present in more than 1 patient (Table 3b). Three were non-synonymous changes (rs3729986—V158M—carried by R810Q, rs34580776—R326Q—carried by Q1233X, and rs11570082—A522T—carried by V771M) with a potential functional effect on the protein. Six were known synonymous SNPs. Only one, a delC in intron 4, was a novel SNP found in multiple patients (Table 3b).

### Prediction of the missense mutations' effect on the protein, and genotype/phenotype correlation

To obtain more functional information on those mutations and polymorphisms that caused an amino acid change, we used the in silico bioinformatic tool PolyPhen (http://genetics.bwh.harvard.edu/pph/) to functionally classify the novel variations detected. PolyPhen categorizes variations to indicate the predicted effect on the protein as benign, possibly damaging, or probably damaging.

#### MYH7

N444S, D1652Y, and L517M were predicted to be damaging for the function of the protein, whereas M932K and S1491C were defined as benign (Table 4a). The novel non-synonymous SNP Q1398L was also predicted as being damaging.

**Table 4:** Prediction of the Functional Effect of the Detected Genetic Variations* With Polymorphism Phenotyping (PolyPhen)

Gene sequence variation	Protein sequence variation	Functional prediction	PSIC score difference
Panel (a) *MYH7*			
c.1331 A>G exon 12	N444S	Probably damaging	2.484
c.4193 A>T exon 29	Q1398L	Probably damaging	2.470
c.4954 G>T exon 33	D1652Y	Possibly damaging	2.660
c.1549 C>A exon 13	L517M	Possibly damaging	1.723
c.2795 T>A exon 21	M932K	Benign	0.864
c.4472 C>G exon 30	S1491C	Benign	0.155

Gene sequence variation	Protein sequence variation	Functional prediction	PSIC score difference
Panel (b) *MYBPC3*			
c.2002 C>T exon 19	R668C	Probably damaging	2.572
c.3251 T>C exon 28	L1084P	Probably damaging	2.346
c.1090 G>A exon 11	A364T	Possibly damaging	1.760
c.2654 C>T exon 24	T885M	Possibly damaging	1.983
c.2429 G>A exon23	R810Q	Possibly damaging	1.947
c.472 G>A exon4	V158M (rs3729986)	Benign	1.051
c.2311 G>A exon22	V771M	Benign	1.435
c.1564 G>A exon15	A522T (rs11570082)	Benign	0.206
c.1624 G>C exon 15	E542Q	Benign	1.408
c.1813 G>C exon17	D605H	Benign	0.055
c.1373 G>A exon14	R458H	Benign	0.185

* Mutations and SNPs that cause an amino acid change; PSIC, position-specific independent counts—tells if the amino acid replacement may be incompatible with the spectrum of substitutions observed at the position in the family of homologous proteins.

#### MYBPC3

According to PolyPhen software, R668C, L1084P, A364T, T885M, and R810Q have a deleterious effect on the protein, whereas V771M, E542Q, D605H, and R458H seem to be tolerated. In addition, the predicted effect of the two known non-synonymous SNPs rs3729986 and rs11570082, which generated V158M and A522T, respectively, were classified as benign (Table 4b).

#### Genotype/phenotype correlation

Even though our clinical database is detailed, establishing significant correlations between genotype and phenotype was limited by the small sample size. We found only poor correlations between the predicted effect of each novel mutation on the protein, as established by PolyPhen software, and the clinical profile of patients carrying those mutations (Supplementary Tables S4 and S5). However, we found that among the novel mutations in *MYBPC3*, R458H seemed to be associated with a clearly benign course of disease, and that among those in *MYH7*, the patient harboring D1652Y (patient ID 183) had an early onset of the disease and a family history of sudden death, and the patient with the L517M mutation (patient ID 248) evolved to the end-stage phase of the disease.

Moreover, we observed that patients harboring a mutation in either *MYH7* or *MYBPC3* were tendentially younger than those without a mutation on one of these genes [respectively, 33 ± 16 years old (*P* = 0.10) and 33 ± 18 years old (*P* = 0.08) vs. 42 ± 18 years old], suggesting that the disease might have an earlier onset in these cases. More interestingly, patients harboring a mutation in *MYBPC3* were less prone to LV outflow tract obstruction [2/19 (10.5%) vs. 36/106 (37%); *P* = 0.032]. Familial presentation was more frequent in patients harboring a mutation in *MYH7* (78% vs. 38%; *P* = 0.031). On the contrary, we did not observed any correlation between a mutation in either *MYH7* or *MYBPC3* and other clinical variables, including extension and severity of LV hypertrophy and history of syncope or sudden death.

## Discussion

Here we report an analysis of the entire coding and junction regions of β-myosin heavy chain and cardiac myosin binding protein—which are known to be the genes most frequently implicated in HCM pathogenesis (Keren et al., 2008)—in a cohort of 136 Italian patients (125 of which were unrelated) presenting with HCM. We used DHPLC to identify variations, which we then characterized qualitatively using bi-directional sequencing. We found 3 novel mutations in *MYH7* (2.4%) and 8 novel mutations in *MYBPC3* (6.4%) out of a total of 6 (4.8%) and 18 (14.4%) distinct mutations, respectively. Mutations in *MYH7* were harbored by 7.2% of patients and in *MYBPC3* by 15.2% of patients.

The low number of mutations we found in these two genes was striking compared to published reports (Keren et al., 2008). Although this finding might throw doubt on the sensibility or suitability of the approached adopted, in our experience the DHPLC conditions we use reach >95% sensibility in detecting genetic variations. Moreover, a similar frequency of mutations was reported in unrelated Spanish patients diagnosed with HCM (Van Driest et al., 2005; Garcia-Castro et al., 2009). A study conducted on a large French population reported higher percentages (Richard et al., 2003), but this discrepancy could be due to genetic heterogeneity or to differences in the ratio of the sporadic to familiar cases in the two studies. Thus, the frequency of sarcomeric mutations might be higher among patients with a family history of the disease. Our findings might also be due to a studied group of subjects that happened to be extremely selective and made up of subphenotypes of mostly (92%) asymptomatic or mildly symptomatic patients. In addition, it was interesting to notice that a majority of the mutations found were novel: this was another aspect that made us suppose that the group of patients studied was more selective compared to other studied HCM populations.

Because none of the genetic variations we found were present in our control group of 384 chromosomes, we concluded that they can be regarded as disease-causing mutations. We classified the missense mutations in terms of severity, taking into consideration the amino acid position, the frequency, and the chemical property differences of the amino acid changes, using PolyPhen software (Table 4). However, it should be considered that not all mutations that cause amino acid changes have a deleterious effect, and that not all silent mutation are benign. In fact, an appreciable affect on the protein can be registered if chemical properties of the new and original amino acids are significantly different (de la Chaux et al., 2007; Gorlov et al., 2008) or if the location where the substitution occurs is important (i.e., a site recognized for splicing or completely conserved residues in the protein family). An amino acid change might be tolerated when it occurs in a position not important for protein function or structure. Variations in moderately conserved sites, instead, are more likely to generate a diseased phenotype altering the clinical manifestation, whereas a variation occurring in a more polymorphic region might impact less negatively on the protein's function. However, in the latter case even if an overt disease phenotype is not generated, the variation might be clinically important if it modulates the severity of an overlying disease or the response of a patient to a given therapy.

Besides substitutions in coding regions, we considered also those that resided in the junction boundaries: these are potentially important if they are responsible for alternative splicing events generating a deficient protein or a shortened 3'UTR. However, at the moment we cannot exclude the possibility that the mutations found are not solely responsible for disease. Only an extensive screening of other genes related to cardiac function can define this aspect. In fact, we did not find a mutation in *MYH7* or in *MYBPC3* in 77.6% patients, suggesting that other genes—sarcomeric-related or otherwise—must be altered. Moreover, we need to pay attention to the number of genetic variations (SNPs) that might play, in concert with mutations, a pivotal role in the development of the disease.

Polymorphisms have a high frequency in the genome: more than 2 million are thought to reside in the human genome, approximately 100,000 of which might affect protein structure or expression (Marian, 2001). We detected multiple synonymous SNPs in a majority of patients, and found non-synonymous SNPs in four patients. Although SNPs very likely complicate the genetic scenario also of HCM, we can only speculate at the moment which might have an effect on the disease, such as in defining drug response or clinical manifestations (Brookes et al., 2000). For example, we found two unrelated patients carrying the same *MYBPC3* K754EfsX78 mutation: at the time of diagnosis, both were asymptomatic, but later on one required an implantable defibrillator while the other did not, suggesting that other genetic variations were pivotal in the natural history of the disease in these patients. Although we are aware that the patients might harbor mutations in other genes related to HCM, the presence in one of the patients of a delC in an intron of *MYBPC3* may be responsible for the different clinical profiles.

Finally, we did not identify any significant differences in the phenotypes manifested by the various mutations of the two genes studied. This is in agreement with literature on unrelated patients (Richard et al., 2003). We are tempted to speculate, therefore, that HCM is probably more similar to a complex disease than to a single-gene disease. Thus, it would be difficult to use partial genetic information to stratify-risk unrelated patients. Novel genetic and environmental causes of HCM must be uncovered in order to fully determine the pathogenic mechanisms taking place in patients with HCM.

The findings obtained here have convinced us of the necessity of adopting a broader methodology, for example, 454 Roche sequencing (Ronaghi et al., 1998; Margulies et al., 2005; Bordoni et al., 2008) or “whole-exome sequencing” (Ng et al., 2010), to carry out a more extensive investigation on a larger group of genes and patients. Sequencing data will be filtered and the selected variations will be validated with a custom chip (GoldenGate, Illumina, San Diego, CA) (Lynch et al., 2009). We expect to identify further causative mutations and a list of SNPs that may be involved in determining clinical events in the development of HCM.
