# Supplementary material for: Unexpectedly Low Mutation Rates in Beta-Myosin Heavy Chain and Cardiac Myosin Binding Protein Genes in Italian Patients With Hypertrophic Cardiomyopathy
Source: J Cell Physiol. 2011 Feb 1;226(11):2894–900. doi: 10.1002/jcp.22636 (PMC3229838; doi:10.1002/jcp.22636)
Supplement: Supplementary file 1 [file jcp0226-2894-SD1.doc]

**Supplementary Table S1. Primer design and DHPLC conditions for *MYH7*.**

| **Exon** | **Primers** | | **Product size**  **(bp)** | **Tm (°C)** |
| --- | --- | --- | --- | --- |
|  | **Forward** | **Reverse** |  |  |
| 1 (5'UTR) | catatatacagcccctgagacca | cttatcccagagtaaagcctccag | 112 | 60 |
| 2 (5'UTR) | ctttccaccttgttttggaagag | tgcccagtcttactagattttcaa | 228 | 60 |
| 3 | tcttgactcttgagcatggtgcta | tctgtccacccaggtgtacaggtg | 381 | 60 |
| 4 | aggaaggagggaaagcccaggctg | tctgcatgcactcaatctgagtaa | 380 | 60 |
| 5 | actggcaagtcactgctcct | cagttcccttcaggaagacct | 226 | 60 |
| 6 | gagcatcctgtgcagctcctga | gaggctgagtctatgcctcgggg | 175 | 60 |
| 7 | cttgctggtctccagtagtattgt | ctgcggtacaggaccttggagggc | 198 | 60 |
| 8 | gccctccaaggtcctgtaccgcag | gtccaagtcccaaggccaaggtca | 200 | 60 |
| 9 | gacaactcctcccgcttcgtg | aacagagggagggaggggagag | 281 | 60 |
| 10 | ccttttgcttgctacatttatcat | gccacaagcagaggggaccag | 252 | 60 |
| 11 | ctgcttcctcaggccatgtgctgt | gtttgcccctcactgccaatcc | 242 | 60 |
| 12 | cacagggattaaggagacaagttt | ttacagctgccccaagaatc | 273 | 60 |
| 13 | agtcatctctttaccaactttgcta | attatcatctgaagatggacccacc | 186 | 60 |
| 14 | caagttcactcttcccaacaaccct | atgtgggagcgagtgagtgattgtt | 258 | 60 |
| 15 | actcacacccactttctgactgctc | gaattcaggtggtaaggccaaagag | 247 | 60 |
| 16 | ataactgtactcagagctgagccta | tccatcccactgagtctgtaaacct | 578 | 60 |
| 17 | gcaaatgccagcaaggatgtaaag | agagaagggagatgggaagtaa | 359 | 60 |
| 18 | ccttccttcttctcctctcttctt | agatgtcctaggaggtcctgttc | 237 | 60 |
| 19 | acaaagccaggatcagaacccaga | ccctgttctatgagctctggtg | 229 | 60 |
| 20 | gaattctatgggcagagcagat | gcatcagaggagtcaatggaaaag | 249 | 60 |
| 21 | taggctgttacccttcctaaggta | gcctctgaccctgtgactgcagtg | 374 | 62 |
| 22 | aggctcagcactcctttcaat | tgtgcagggaggtgcagggttgtg | 366 | 62 |
| 23 | ctgcaagaatgaggaccttacc | atggtctgagagtcctgatgagac | 355 | 62 |
| 24 | accctaaaggagatgggattct | tctgggcacagatagacatggcat | 264 | 60 |
| 25 | ttaccaagtcctgaggtaactgaa | cttgggtctgcttgtactgttatg | 248 | 62 |
| 26 | actctttacctgtatcattaccat | gtgaacaggacaccctagagga | 271 | 60 |
| 27 | gtgggtctgagccctttgt | ggaggaggaagttggaggag | 452 | 62 |
| 28 | ccccaacatccatcatataactct | tctggagagactctgtgtctgtgt | 280 | 60 |
| 29 | agaggagtgctgatctagaatggt | gatttgatgcaaggctagtcagt | 218 | 62 |
| 30 | gttgctttatggagaaagctgaac | ttgtcaaacactagctaagcatcg | 360 | 60 |
| 31 | catccacaccctccatcctc | gcctctcactgaacccctcat | 300 | 60 |
| 32 | tcctgagacagaccctggac | tggcaccatatgggaacact | 296 | 60 |
| 33 | cctcaaccgagttaccgtgt | acagtgaacaaaacggacaaag | 250 | 60 |
| 34 | ttcgtatccatgattagtgagcag | tggtgcctgtatcaagacactact | 463 | 62 |
| 35 | ctctgacaggtgcctttagtgaa | gcaggaaaagcattgagcatcta | 372 | 60 |
| 36 | atagatgctcaatgcttttcctg | gtctggtcaagtcctcacacact | 258 | 60 |
| 37 | agtgtgtgaggacttgaccagac | attctcctcagctggttgtcact | 395 | 60 |
| 38 | cttctatgactgtgccatcttcac | ggttctcagactcctggcttg | 221 | 60 |
| 39 | caagccaggagtctgagaacc | gtgactagcaaagcccaaaag | 224 | 60 |
| 40 (3’UTR) | caataccatctctccaaggactg | ctcagggcagtgaagaagagtc | 215 | 60 |

**Supplementary Table S2. Primer design and DHPLC conditions for *MYBPC3*.**

| **Exon** | **Primers** | | **Product size**  **(bp)** | **Tm (°C)** |
| --- | --- | --- | --- | --- |
|  | **Forward** | **Reverse** |  |  |
| 1(5'UTR) | gacctcagctctctggaattcatc | gctcagaggccacgtcctcgtcaa | 311 | 60 |
| 2 | gggtgcacgctccaaccag | gcagcccaaacctcagggaag | 353 | 60 |
| 3 | tggaggggtctctggttagtgg | gcttttgagacctgccctggac | 236 | 60 |
| 4 | gggcacctgcggtcccagctaact | acctgcaaaggcagggcgacag | 350 | 60 |
| 5 | gtgggtgagtgtgagctgctgt | ctctgtgtgccttgtgccttctag | 317 | 60 |
| 6 | ggccactcccagtctcctttaa | actcatgtctggatgggacgag | 313 | 60 |
| 7 | gcttctcaaacggccccctctg | aagggcctcagactccagcactg | 163 | 60 |
| 8 | gggcggagctgaagtcagtgg | gtctgcggatggtgcaggtag | 149 | 60 |
| 9 | cctgctcctaatccctttccagtc | ctcaccagctgccccaggaact | 150 | 60 |
| 10 | ctgtcagagcttaggagggataag | atgagggtgctgtgctatgtt | 264 | 60 |
| 11 | ccagggggctgcagtcttg | ctaagccggactccgctcttt | 370 | 60 |
| 12 | ggcggcacagaggggattg | accggcaggagcaaaaggatg | 402 | 60 |
| 13 | ggtgtccgcagctttcctg | ggtgagcatgagggttggc | 241 | 60 |
| 14 | acctgaggatgtgggaacct | caagtgctgtggcctcttct | 245 | 60 |
| 15 | cagaagaggccacagcact | tccaagccctaaagcctca | 250 | 60 |
| 16 | acaggcacacgtgttttcac | cagtctccacctgtcccatc | 345 | 60 |
| 17 | gaccctggctggggtatctg | cgacccaccctaccctgga | 250 | 60 |
| 18 | tgcctttgcccccgtgctacttg | ccacttttgatccttgctcttc | 175 | 60 |
| 19 | tcctcctggctctcccgtttctct | ggttccacacacccatcttatag | 240 | 60 |
| 20 | gctcctctgctccctacttcc | atggccatcagcacacttcac | 310 | 60 |
| 21 | agggcctctggggtctgacttg | ggctgcccctctgtgttctcca | 256 | 60 |
| 22 | cggttagttggagtgggaagg | cttccctcggatctgtttgg | 258 | 60 |
| 23 | ccgagggaaggtggtgtgg | tctgtaaaatgcggctgagtatcc | 404 | 60 |
| 24 | cttgctcagacccctctctg | tacaggtgaatctgctcaatgg | 457 | 60 |
| 25 | tcagaggagtgggcagtgggagtg | ctggggtgtcaatggcgggtctt | 292 | 60 |
| 26 | ccctcacttagctacccactct | gatgggaacaacacactatagcc | 182 | 60 |
| 27 | tgcggccggcccttggagt | gaaacaagggggctcaagg | 285 | 60 |
| 28 | gaggcgtggtgacccaactg | cacggtgaggacagtgaagggtagc | 263 | 62 |
| 29 | ggccgcagctacccttcac | aggcccctctccctgttcc | 392 | 60 |
| 30 | ggcctctcggtaccaagtcctgtc | caacgtcggggcctgtgagc | 232 | 60 |
| 31 | gatggcttccctccctctc | ccgcccgctcttcccatctc | 270 | 60 |
| 32 | cacagtgacatggcctcctcttct | gcccctacagcctcccatttact | 159 | 60 |
| 3'UTR | cctccattcactcgtaagataacc | atttttatgaaaacaggcacacc | 382 | 60 |

**Supplementary Table S3. Clinical characteristics of HCM patients carrying *MYH7* and *MYBPC3* mutations.**

| **Clinical features** | ***MYH7***  **(n= 9)** | ***MYBPC3***  **(n= 19)** |
| --- | --- | --- |
| Men (%) | 5 (55.5) | 14 (73.7) |
| Age at study | 48.3 ± 16.1^a^ | 48.0 ± 17.6^a^ |
| Age at diagnosis | 33.0 ± 18^a^ | 35.1 ± 17^a^ |
| Family history of HCM (%) | 7 (77.7) | 10 (52.6) |
| Family history of SCD (%) | 5 (55.5) | 5 (26.3) |
| NYHA III-IV functional class (%) | 0 (0) | 1 (5.3) |
| Chest pain (%) | 4 (66.6) | 5 (26.3) |
| Syncope (%) | 2 (22.2) | 2 (10.5) |
| NSVT (%) | 2 (22.2) | 2 (10.5) |
| Atrial fibrillation^b^ (%) | 0 (0) | 3 (15.8) |
| **Electrocardiogram** | | |
| LVH (%) | 5 (55.5) | 10 (52.6) |
| Q wave (%) | 5 (55.5) | 7 (36.8) |
| T wave inversion (%) | 6 (66.6) | 8 (42.1) |
| **Echocardiographic features** | |  |
| LVOT obstruction (%) | 5 (55.5) | 1 (5.3) |
| Left atrium (mm) | 45.3 ±6 | 43.8 ±8 |
| Maximum LVWT (mm) | 21.1 ± 7.5^a^ | 20.8 ± 4.6^a^ |
| Localization of hypertrophy: |  |  |
| - Anterior septum (%) | 8 (88.9) | 18 (94.7) |
| - Posterior septum (%) | 1 (11.1) | 0 (0) |
| - Lateral wall (%) | 0 (0) | 1 (5.3) |
| - Apical wall (%) | 0 (0) | 0 (0) |
| LVEF (%) | 5 (55.5) | 1 (5.3) |
| **Interventions and events** | |  |
| ICD (% ) | 2 (22.2) | 4 (21.0) |
| Myotomy/myectomy (%) | 0 (0) | 1 (5.3) |
| Alcohol septal ablation (%) | 0 (0) | 0 (0) |
| End-stage evolution (%) | 1 (11.1) | 0 (0) |
| Stroke (%) | 0 (0) | 1 (5.3) |
| ICD intervention (%) | 0 (0) | 1 (5.3) |

^a^ mean±SD; SCD, sudden cardiac death; NYHA III-IV, New York Heart Association class III-IV (classification of heart failure); NSVT, non-sustained ventricular tachycardia; ^b^either paroxymal or permanent; LVH; left ventricular hypertrophy; LVOT, left ventricular outflow tract; LVWT, left ventricular wall thickness; LVEF, left ventricular ejection fraction; ICD, implantable cardioverter defibrillator.

**Supplementary Table S4. Clinical features of HCM patients with *MYBPC3* mutations**

| **Mutation** | **ID** | **Age at study/**  **gender** | **Age at diagnosis** | **FH**  **SCD** | **NYHA III-IV** | **Chest pain** | **Syncope** | **NSVT** | **AF** | **LVOT**  **obstr.** | **Max**  **LVWT** | **Loc.** | **LVEF (%)** |
| --- | --- | --- | --- | --- | --- | --- | --- | --- | --- | --- | --- | --- | --- |
| **A364T** | 233 | 51/F | 27 | Y | N | N | N | N | N | N | 27 | ANT | 69 |
| **D605H** ^§ N^ | 180 | 31/F | 31 | Y | N | N | N | N | N | N | 16 | LAT | 65 |
| **D605H** ^§ F^ | 208 | 75/M | 61 | Y | Y | N | N | N | Y | N | 18 | ANT | 74 |
| **D605H** ^§ S^ | 186 | 15/M | 15 | Y | N | N | N | N | N | N | 24 | ANT | 65 |
| **E542Q** | 244 | 58//M | 40 | N | N | Y | N | Y | N | N | 21 | ANT | 60 |
| **2309-2** ex 22  rs1052373 | 234 | 22/M | 15 | N | N | Y | Y | N | N | N | 16 | ANT | 65 |
| **2905+1** (ex25) | 157 | 28/M | 15 | Y | N | N | N | Y | N | N | 21 | ANT | 65 |
| **Q969X** | 188 | 53/F | 40 | Y | N | N | N | N | N | N | 30 | LAT | 65 |
| **R943X** | 220 | 42/M | 36 | N | N | N | Y | Y | N | N | 22 | ANT | 55 |
| **Q1012X**  rs11570078 | 181 | 23/M | 16 | N | N | N | N | N | N | N | 25 | POST | 65 |
| **Q1233X**  rs3729948  rs34580776  rs3729936  rs3729953 | 251 | 28/M | 20 | N | N | Y | Y | Y | N | N | 32 | ANT | 60 |
| **M949I fsX100**  rs 3729948  rs 3729953 | 278 | 34/M | 21 | N | N | N | N | N | N | N | 18 | ANT | 60 |
| **K754E fsX78** | 208 | 66/M | 35 | Y | N | N | N | N | N | N | 25 | ANT | 69 |
| **K754E fsX78**  del C | 195 | 34/M | 29 | N | N | N | N | N | N | N | 25 | ANT | 72 |
| **K1065Q fsX11**  rs11570078  rs3729948  rs3729936 | 194 | 32/F | 12 | N | N | N | N | N | N | N | 34 | ANT | 65 |
| **T885M** | 265 | 59/F | 55 | N | N | N | N | N | N | Y | 19 | LAT | 76 |
| **R668C** | 214 | 76/M | 69 | N | N | N | N | N | N | N | 20 | ANT | 60 |
| **L1084P** ^#F^ | 185 | 60/M | 54 | Y | N | N | N | Y | Y | N | 19 | ANT | 70 |
| **L1084P** ^#S^ | 177 | 24/M | 24 | Y | N | N | N | N | N | N | 13 | ANT | 70 |
| **V771M**  rs1170082 rs11570078 | 221 | 69/M | 33 | N | N | N | Y | Y | Y | N | 20 | ANT | 60 |
| **R810Q**  rs 3729986 | 270 | 43/M | 32 | N | N | N | N | N | N | N | 22 | ANT |  |
| **R458H**  rs 3729936 rs3729953 rs3729948 | 268 | 60/F | 57 | N | N | N | N | N | N | N | 17 | ANT | 65 |

FHSCD, family history of sudden cardiac death; NYHA III-IV, New York Heart Association class III-IV; NSVT, non-sustained ventricular tachycardia; AF, atrial fibrillation either paroxymal or permanent; LVOT obstr., left ventricular outflow tract obstruction; Max LVWT, maximum left ventricular wall thickness; Loc., localization; ANT, anterior septum; POST, posterior septum; LAT, lateral wall; LVEF, left ventricular ejection fraction; ^§^Related patients; #Related patients: ^N^Nephew; ^F^Father; ^S^Son; F, female; M, male; Y, yes; N, no.

**Supplementary Table S5. Clinical features of HCM patients with *MYH7* mutations**

| **Mutation** | **ID** | **Age at study/**  **gender** | **Age**  **at diagnosis** | **FH**  **SCD** | **NYHA III-IV** | **Chest pain** | **Syncope** | **NSVT** | **AF** | **LVOT**  **Obstr.** | **LVWT** | **Loc.** | **EF (%)** |
| --- | --- | --- | --- | --- | --- | --- | --- | --- | --- | --- | --- | --- | --- |
| **N444S** | 154 | 58/F | 54 | N | N | Y | N | N | N | Y | 17 | Ant. | 74 |
| **M932K** | 201 | 51/F | 34 | Y | N | Y | N | Y | N | Y | 30 | Ant. | 64 |
| **D1652Y** | 183 | 47/F | 22 | Y | N | N | N | N | N | Y | 18 | Ant. | 71 |
| **S1491C**^§D^ | 190 | 28/F | 28 | N | N | N | N | N | N | N | 11 | -- | 70 |
| **S1491C**^§M^ | 199 | 50/F | 42 | Y | N | N | N | Y | N | Y | 22 | Ant. | 75 |
| **S1491C** | 206 | 79/M | 66 | N | N | Y | N | Y | Y | Y | 20 | Ant. | 64 |
| **S1491C** | 293 | 35/M | 33 | N | N | N | N | N | N | Y | 15 | Ant. | 65 |
| **L517M** | 248 | 57/M | 17 | Y | N | N | N | Y | Y | N | 16 | Ant. | 45 |
| **A1051A** | 273 | 25/M | 12 | N | N | N | N | Y | N | Y | 27 | Ant. | 68 |
| **A1051A** | 227 | 33/M | 18 | Y | N | N | N | Y | N | N | 36 | Ant. | 65 |

SCD, family history of sudden cardiac death; NYHA III-IV, New York Heart Association class III-IV; NSVT, non-sustained ventricular tachycardia; AF, atrial fibrillation (either paroxymal or permanent); LVOT obstr., left ventricular outflow tract obstruction; LVWT, maximum left ventricular wall thickness; Loc., localization; ANT, anterior septum; EF, left ventricular ejection fraction; ^§^Related patients: ^D^ Daughter; ^M^ Mother; F; female; M; male; Y, yes; N, no.

Patient n. 154, Ex 12: 1331 A>G – N444S

Patient n. 183, Ex.33: 4954 G>T– D1652Y

**Suppl. Fig. S1.** DHPLC and sequence profiles of novel mutations detected on *MYH7*. Chromatograms and electropherograms for novel missense (A**,** B) and silent (C) mutations.

C


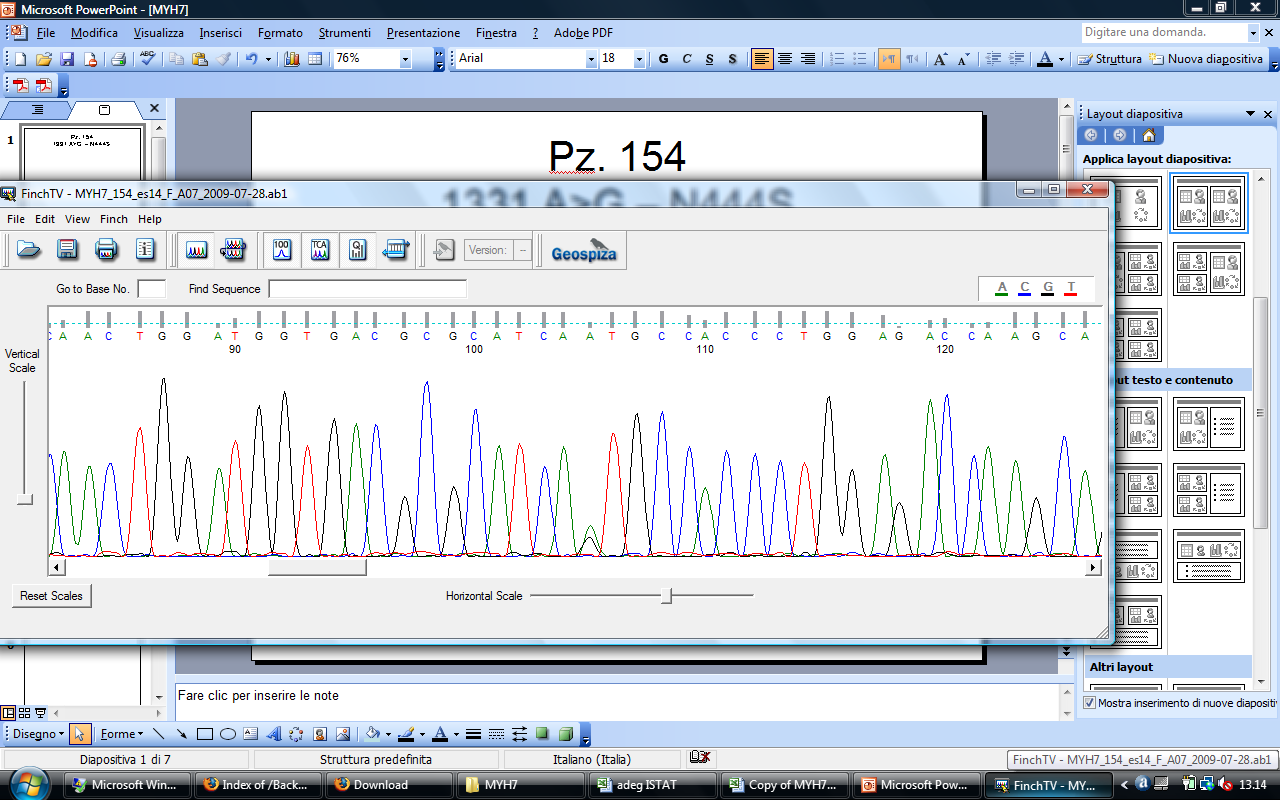

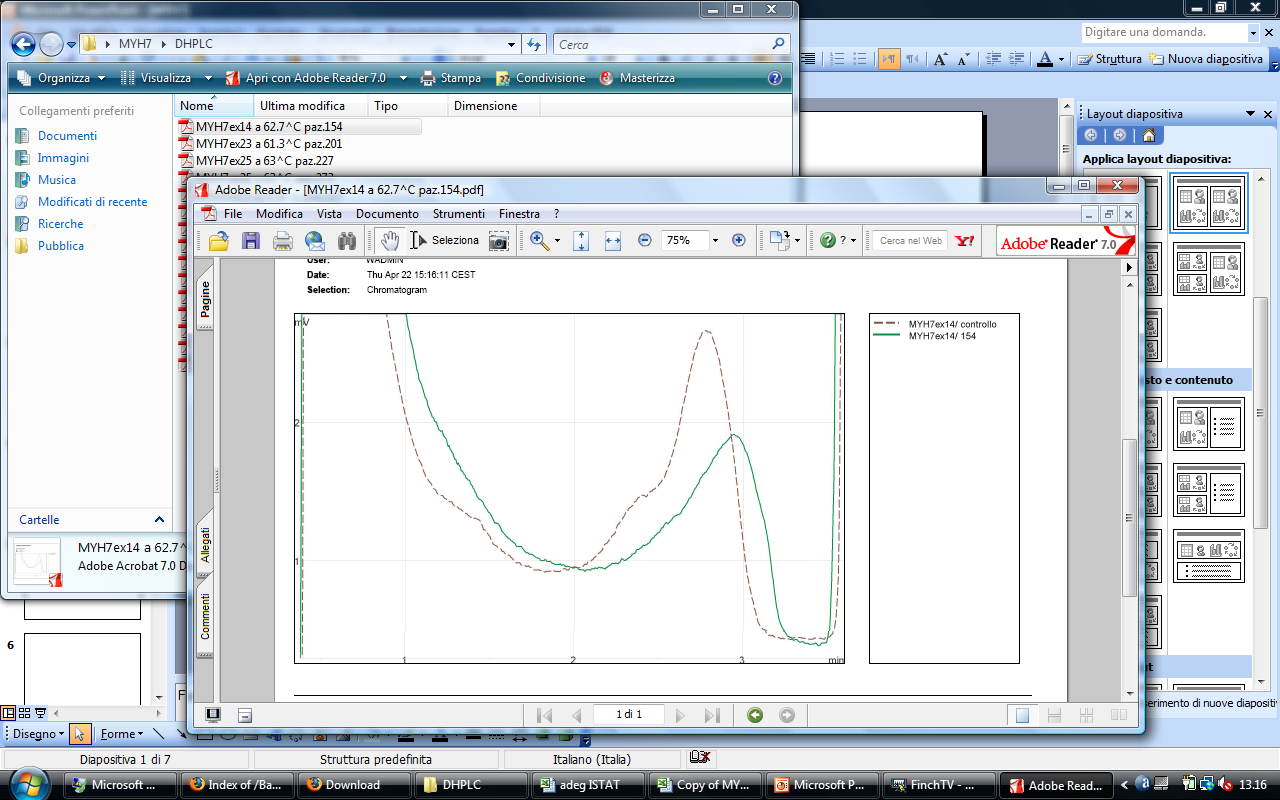

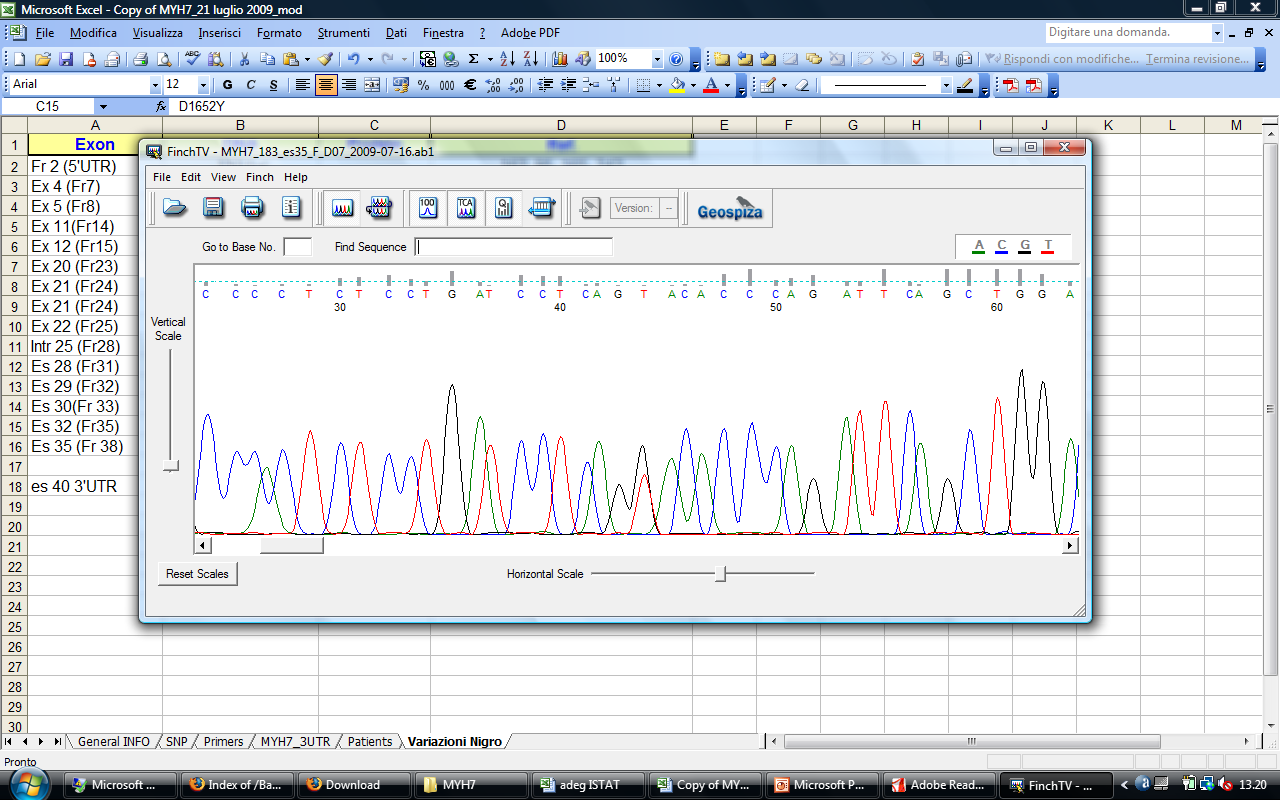


Patient n. 227, 273, Ex 23: 3153 G>A – A1051A


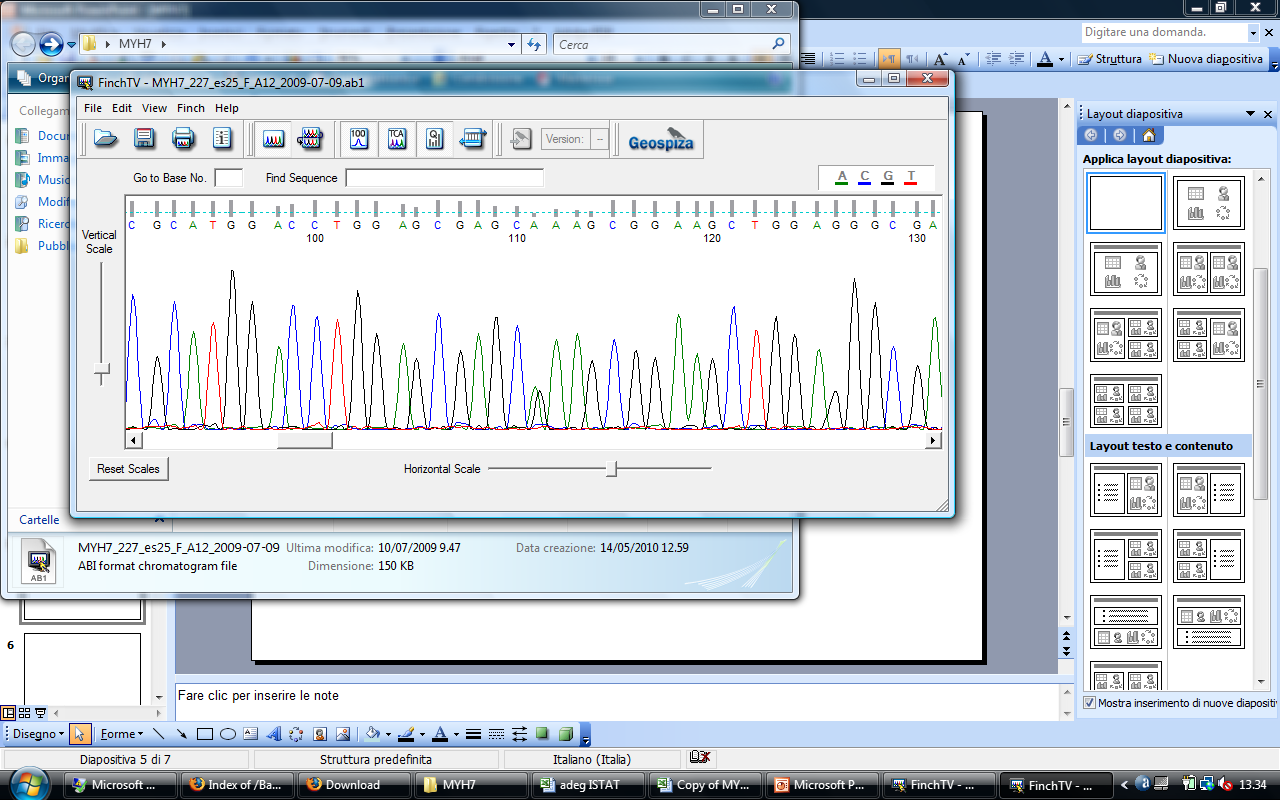


A

B


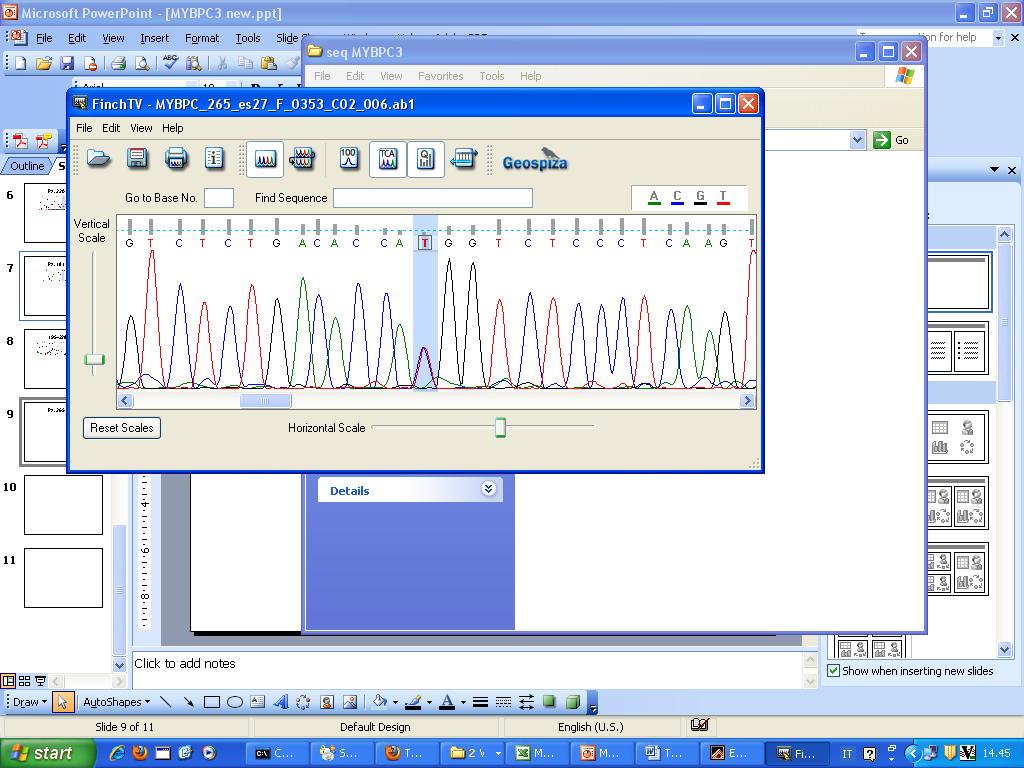

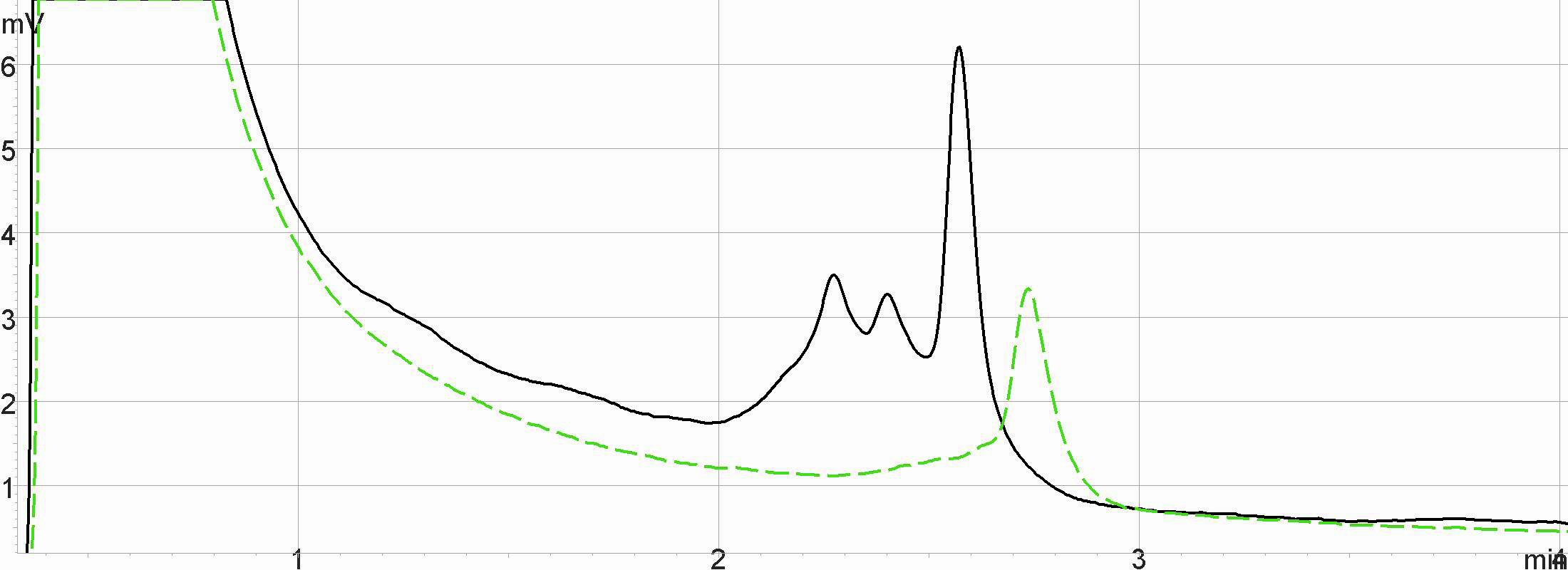

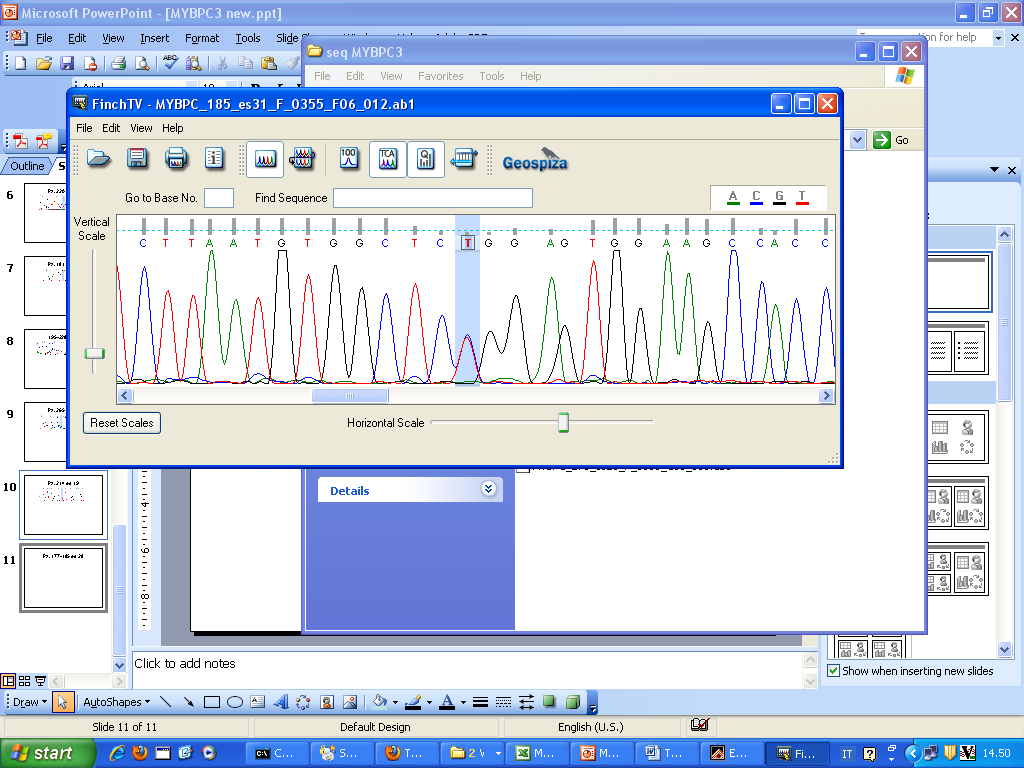

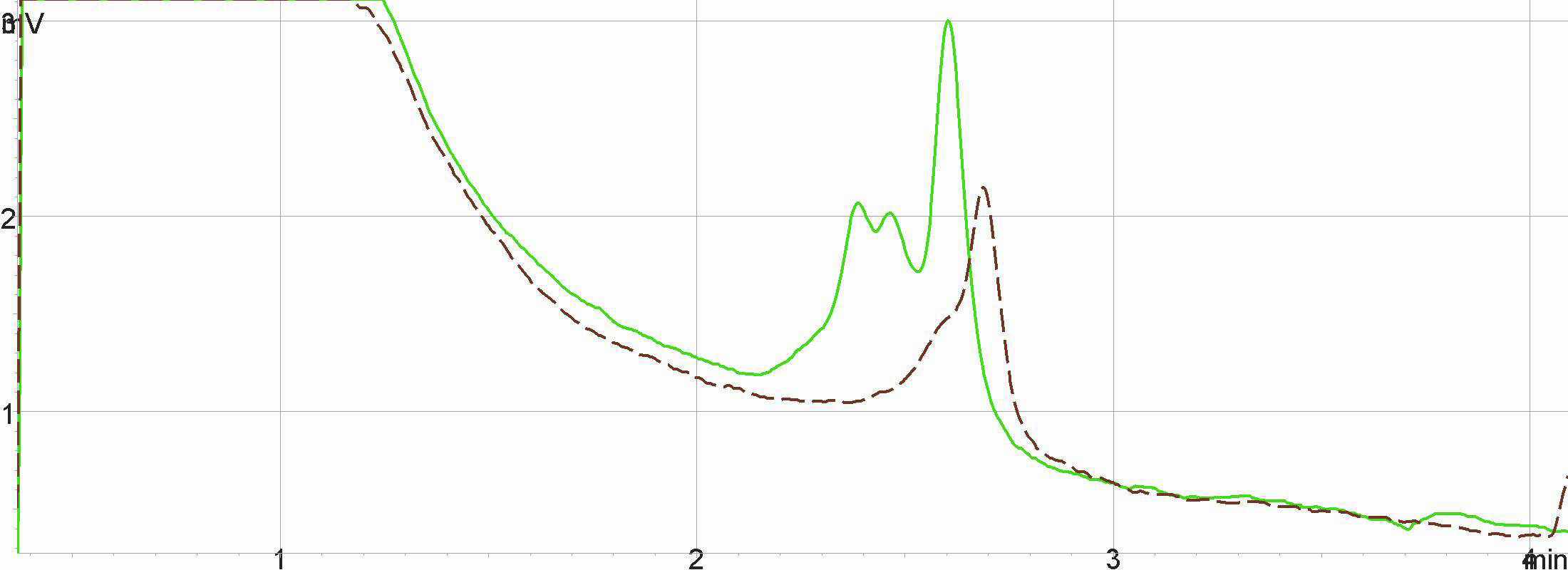

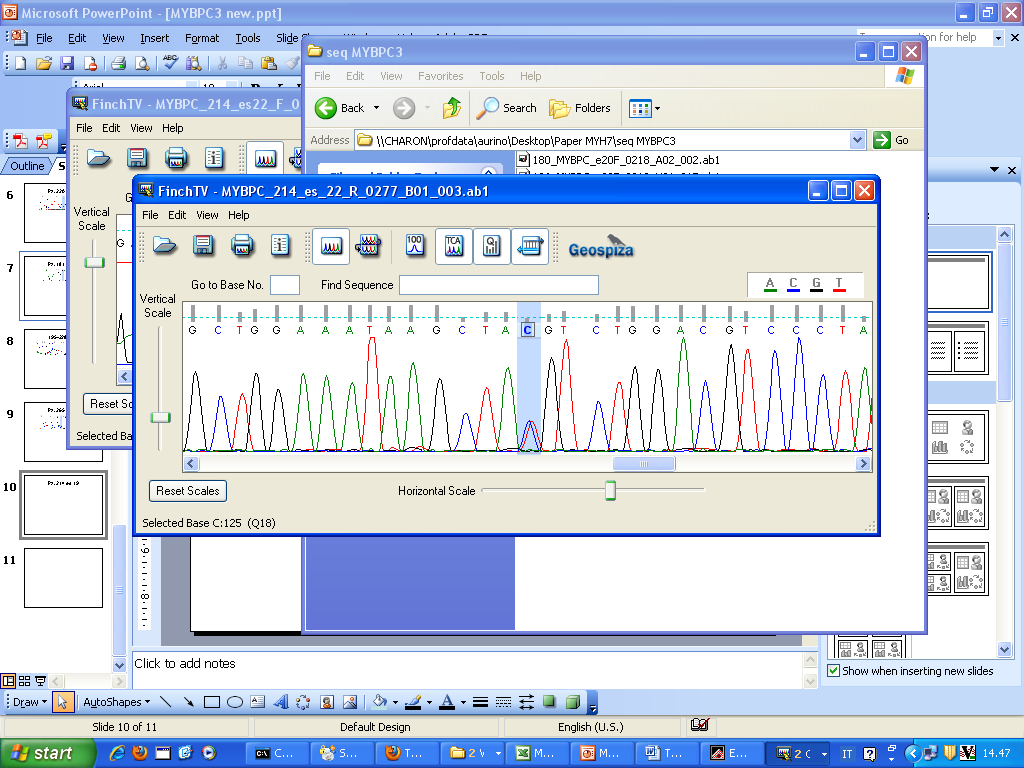

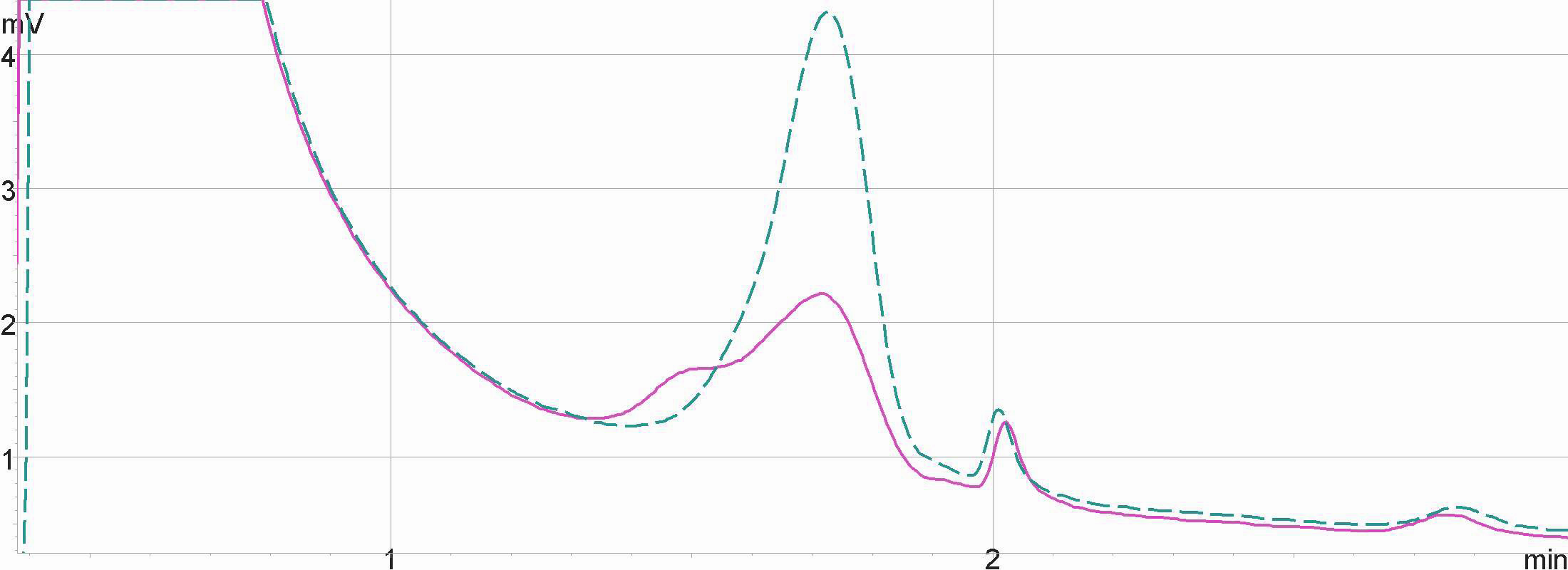

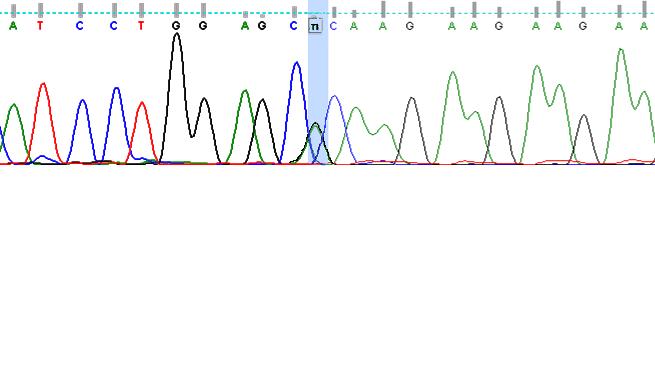

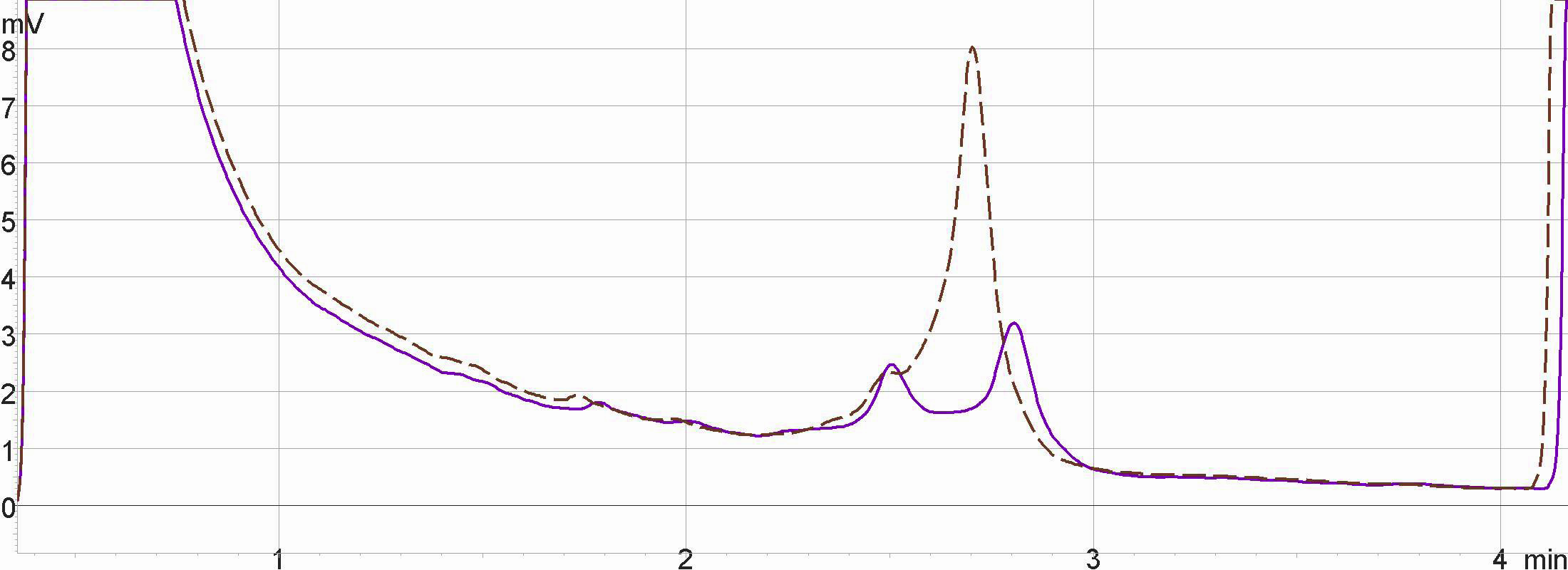

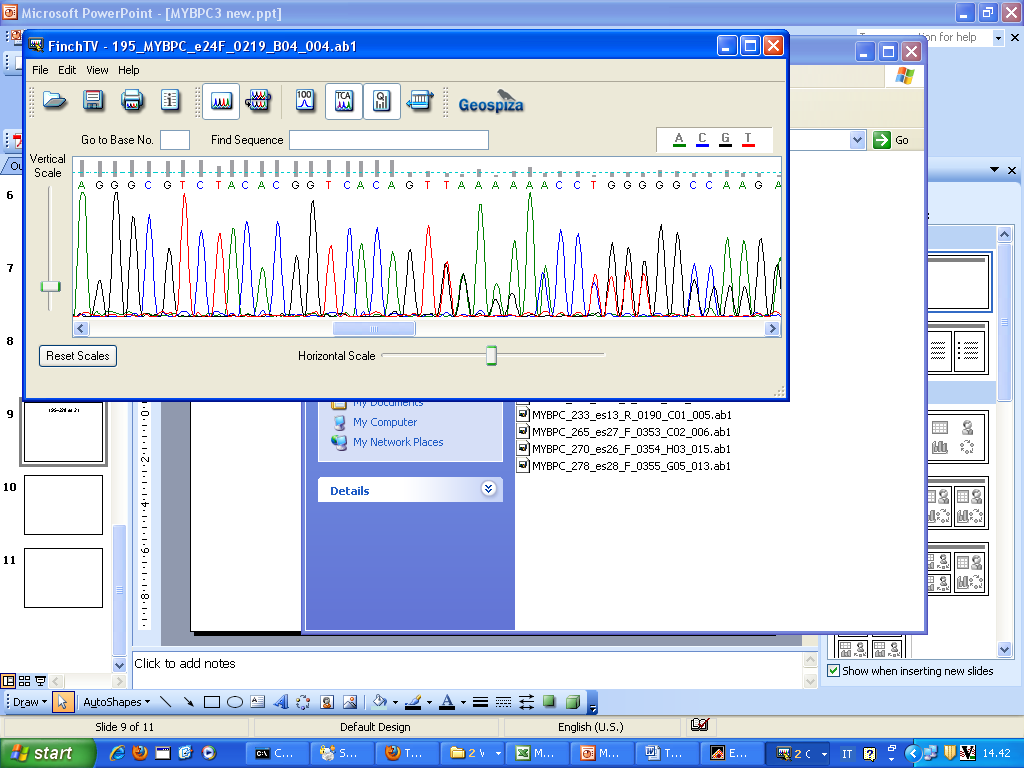

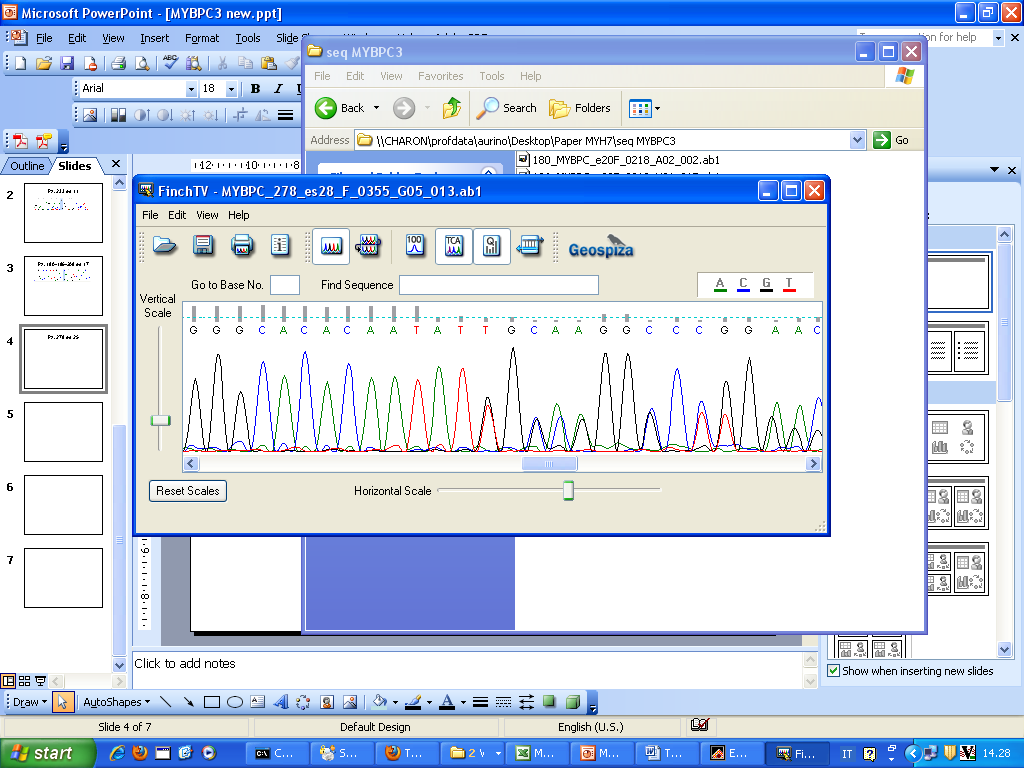

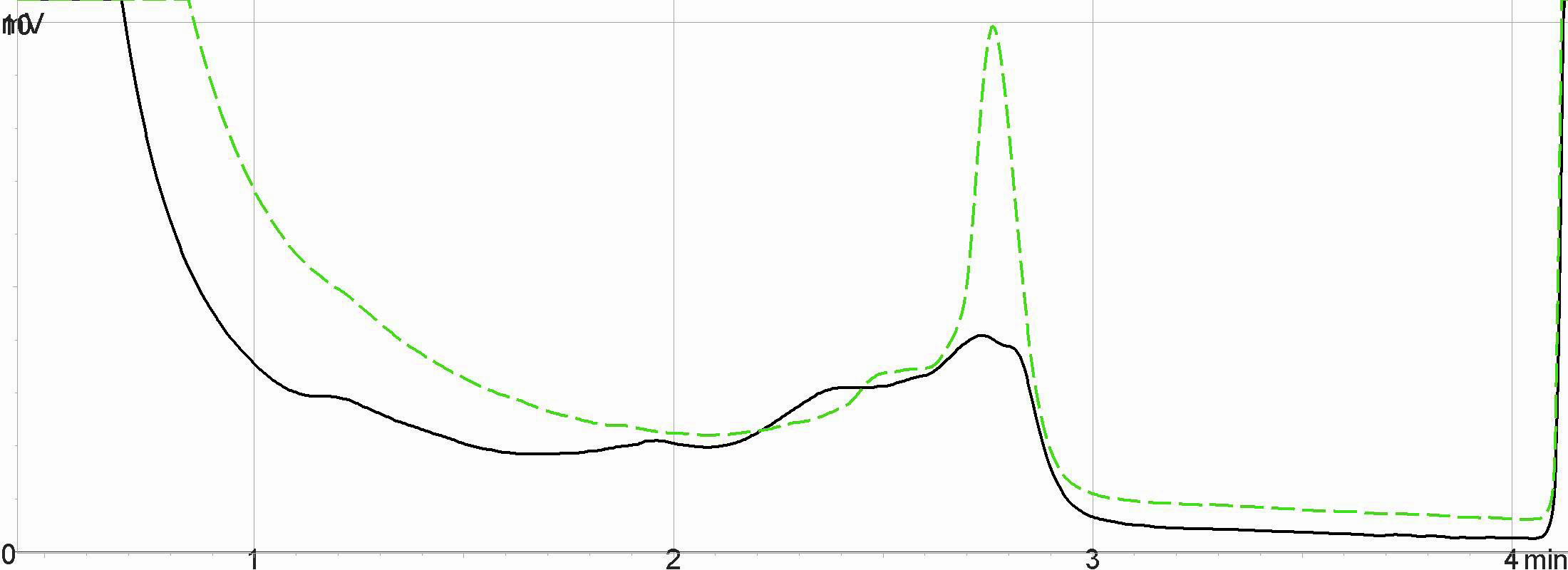

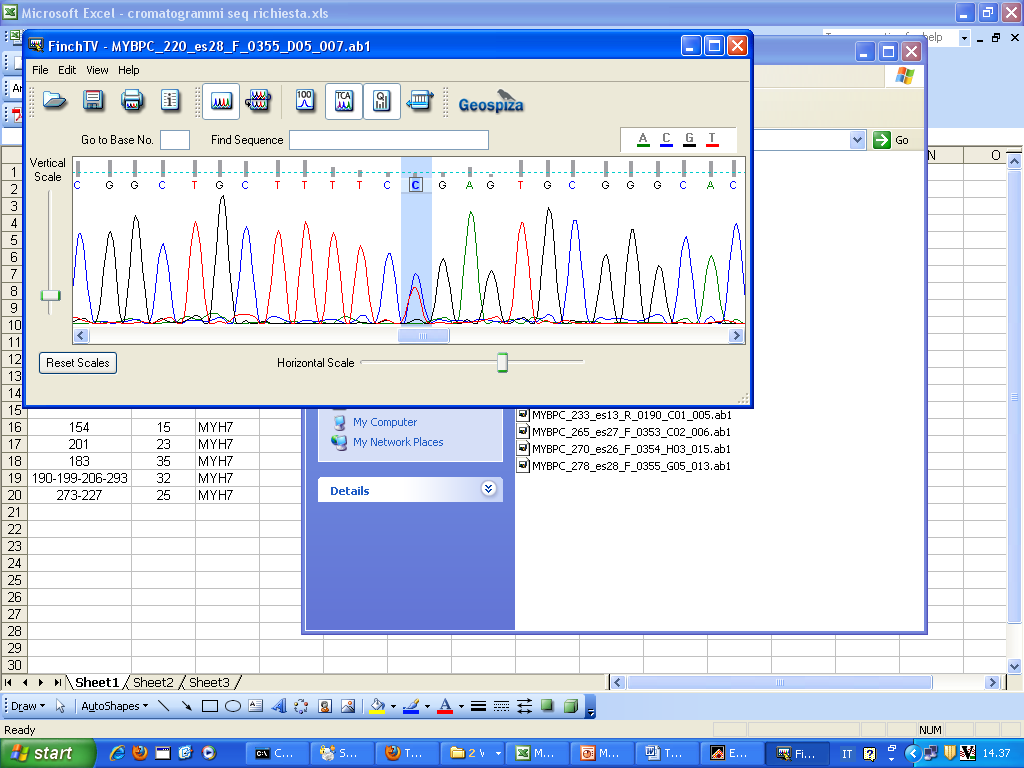

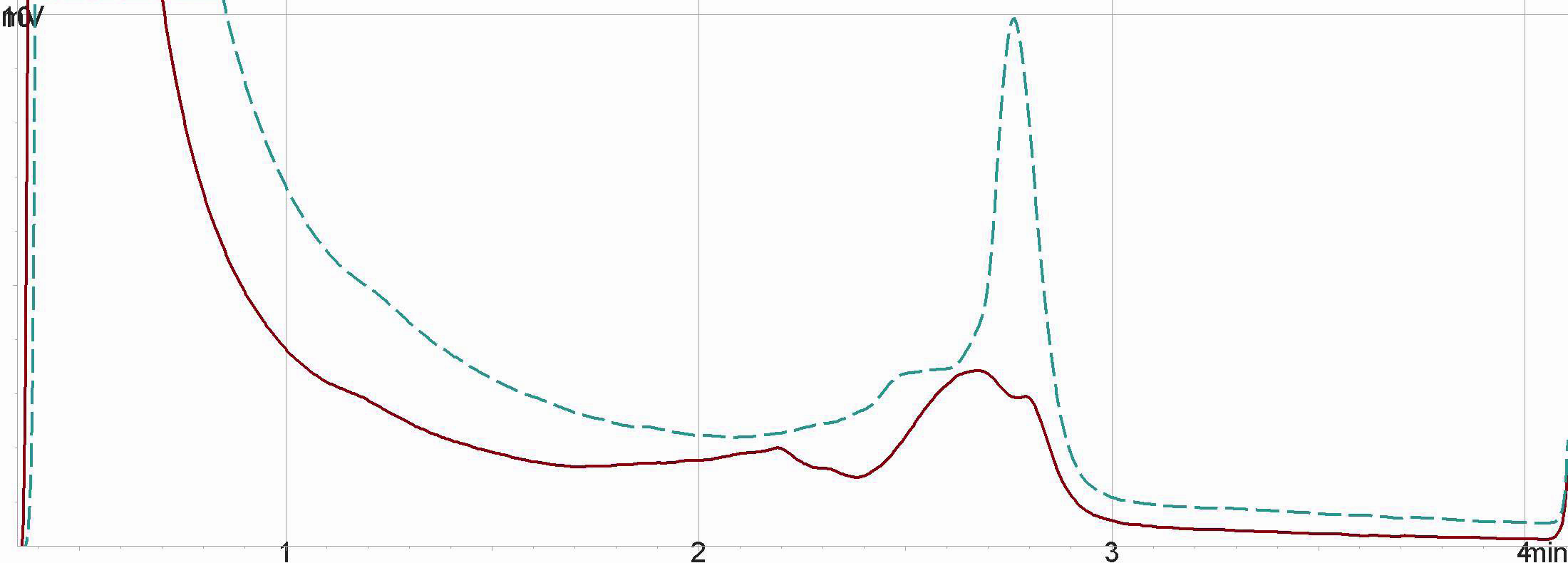

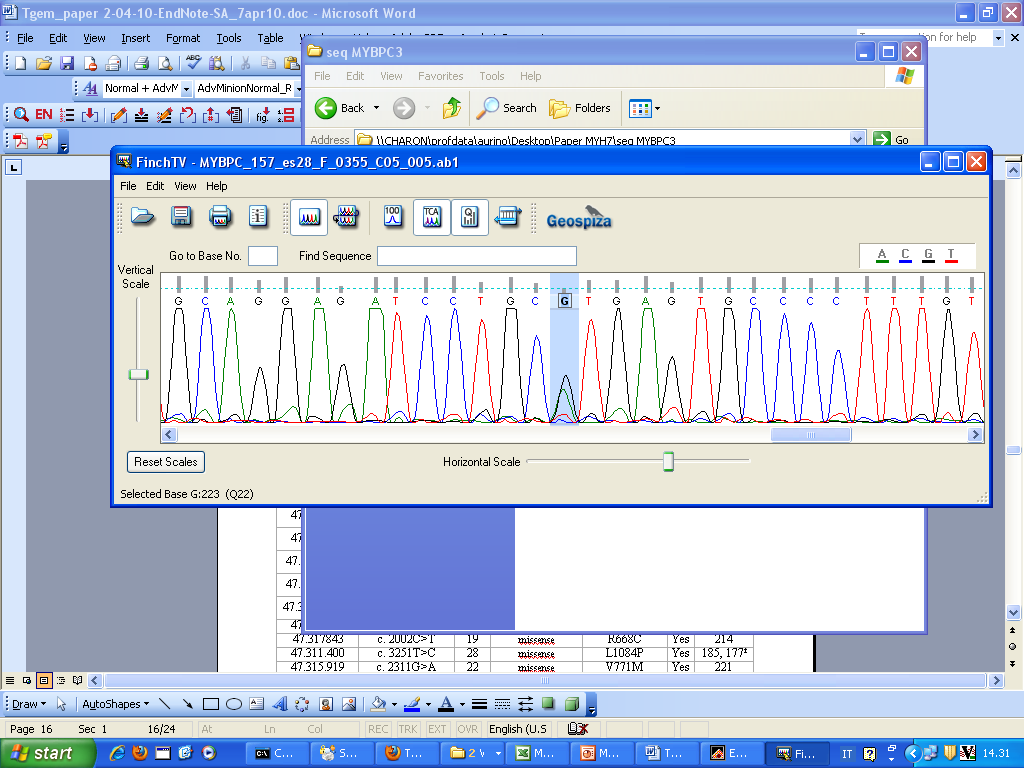


**Suppl. Fig S2.** DHPLC and sequence profiles of novel mutations detected on *MYBPC3*. Chromatograms and electropherograms for a potential splicing alterations (A), a nonsense mutation that generated a stop codon (B), frameshifts that generated stop codons (C, D), and missense mutations (E**,** F, G, H).

Patient n. 157; exon 25

Patient n. 265; exon 24

Patient n. 220; exon 25

Patient n.214; exon 19

Patient n. 278; exon 25

Patient n. 177, 85; exon 28

Patient n.195, 228; exon 21

Patient n.270; exon 23

A

B

C

D

E

F

G

H
